# Global distribution of deep-sea natural products shows environmental and phylogenetic undersampling with potential for biodiscovery

**DOI:** 10.1038/s41598-026-45398-0

**Published:** 2026-07-10

**Authors:** Lucy M. Harris, Gagan Preet, Ria Desai, Ahlam Haj Hasan, Johanne Vad, Guadalupe Bribiesca-Contreras, Rishi Vachaspathy Astakala, Adrian G. Glover, Marcel Jaspars, Jonathan Copley

**Affiliations:** 1 Ocean and Earth Science, University of Southampton, Waterfront Campus, European Way, Southampton, S014 3ZH UK; 2 Marine Biodiscovery Centre, Department of Chemistry, University of Aberdeen, Old Aberdeen, AB24 3UE UK; 3 The Medicinal Chemistry and Pharmacognosy Department, Faculty of Pharmacy, Jordan University of Science and Technology, Irbid, 22110 Jordan; 4 School of Geosciences, University of Edinburgh, Grant Institute, The King’s Buildings, James Hutton Road, Edinburgh, EH9 3FE UK; 5 Ocean BioGeosciences, National Oceanography Centre, European Way, Southampton, S014 3ZH UK; 6 Natural History Museum, Cromwell Rd, South Kensington, London, SW7 5BD UK

**Keywords:** Ecology, Ecology, Ocean sciences

## Abstract

Natural products from marine and terrestrial organisms provide an important resource for medicinal chemistry and drug discovery. Defined as occurring at depths greater than 200 m, most of the ocean, by area and volume, is comprised of deep-sea environments. These contrasting ecosystems exhibit a wide range of temperature, pressure, and environmental chemistry conditions that harbour high phylogenetic diversity. Here we analyse the global distribution of Marine Natural Products (MNPs) from deep-sea organisms, which reveals chronic undersampling of the deep ocean as a reservoir of new and diverse chemical structures and bioactivity. The sources of 2909 compounds and extracts, compiled from published records, show a sampling bias towards benthic and shallower deep-sea habitats, with relatively few from areas beyond national jurisdictions and a concentration of records along geomorphological features that have been the focus of marine scientific interest such as seamounts and hydrothermal vents. The phylogenetic distribution of deep-sea MNPs is dominated by non-metazoan sources (76% of records) and Ascomycota fungi in particular (55%). Polyketides are the most prevalent metabolites in deep-sea MNPs, and cytotoxic and antibacterial properties are the most commonly reported bioactivities. Our analyses highlight a need for systematic sampling and consistent data reporting to explore potential relationships, if any, between, bioactivity, new chemical structures, phylogeny and deep-sea environmental conditions, which could guide targeted biodiscovery in our planet’s largest biome and enable better assessment of the benefits of protecting deep-sea biodiversity from human impacts.

**Supplementary Information:**

The online version contains supplementary material available at 10.1038/s41598-026-45398-0.

## Introduction

The natural world has played a critical role in the discovery and development of pharmaceutical products, derived from compounds found in plants, animals and microorganisms^1^. Continued human population growth—the population of > 60-year-olds being estimated to double (2.1 billion) by 2050—has given rise to increasing demands for health care resources, alongside a higher risk of disease and prevalence of drug resistance ^2,3^. As a result, the vast unexplored genetic diversity of marine ecosystems is a growing focus for the pharmaceutical sector, with ~ 43,000 Marine Natural Products (MNPs) being reported in the last 50 years^4,5^. This is largely owing to new chemical structures, for example with ~ 70% of macrofaunal structural scaffolds endemic to marine organisms^6,7^.

The deep sea constitutes 90% of the global area of the ocean^8^, and the diverse habitats that it contains are likely to host at least 50% of the estimated ~ 2.2 million marine species^9^. As a consequence of heterogeneity in conditions of pressure, temperature, light and chemistry, across numerous environments including, abyssal plains, sea mounts, continental margins, hydrothermal vents, cold water coral reefs, hadal and oxygen minimum zones, the deep sea hosts an extensive reservoir of highly adapted extremophiles^10,11^. The physiological and biochemical adaptations to environmental conditions in deep-sea habitats can be linked to modifications in gene regulation and primary and secondary metabolic pathways, which have the potential to provide structurally unique MNPs^12^. Despite historic undersampling of deep-sea biodiversity^13^, advances in technology and automation and the introduction of increasingly affordable tools capable of recording and collecting deep-sea specimens are revealing the presence and extent of new chemical structures and bioactivity in many species that reside in our deep ocean^14–17^. For example, 80% of the compounds assayed from a study on deep-sea microbes exhibited various bioactivities^18^. However, major gaps in our knowledge of deep-sea biodiversity remain, particularly in the chronically under-represented deep pelagic ocean^13^. Some patterns have emerged, namely that the most common environment in the deep sea, abyssal plains, host low productivity and biomass, but high biodiversity^19,20^. Species diversity is lower in suboptimal deep-sea environments, such as low oxygen zones or hydrothermal vent disturbances^21^. Annelids, arthropods and molluscs in the deep-sea, appear to demonstrate disproportionally high biodiversity, whilst megafauna typically do not trend towards higher biodiversity^22^. Deep-sea microbes offer an underexplored “rare biosphere”, which are more likely to synthesize structurally distinctive enzymes and secondary metabolites compared with their terrestrial and shallow water counterparts^23,24^. It is important to highlight that current ocean sampling is spatially and taxonomically biased towards vertebrate species, the first ~ 100 m of the water column, the northern hemisphere, resource-concentrated areas, certain geomorphological features (canyons, escarpments, and slopes) and certain Exclusive Economic Zones (EEZs)^17,25^.

Several previous analyses of deep-sea natural product data have been restricted to particular taxonomic groups, such as sponges^26,27^, sponge-microbe associations^28,29^, deep-sea fungi^30,31^, actinomycetes^32^, as well as microorganisms^33,34^ and extremophiles in general^35^. Others have focused on specific habitats such as hydrothermal vents^36^, geographical regions^37,38^ and ecosystems^30^. Some studies have focused on individual bioactivities, such as anti-cancer^39^ and antibiotic^40^ properties. A few studies have addressed deep-sea MNPs in general^41–43^, but not specifically exploring relationships between metabolites and the phylogeny or environmental conditions of deep-sea organisms, despite their potential to provide new molecules with potent activities^43^.

Comprehensive inventories of published MNPs, including those sourced from the deep sea, are available annually in the journal Natural Product Reports^44–82^, which led to the development of the online MarinLit MNP (Marine Natural Products) database hosted by the Royal Society of Chemistry since 2013^4^. The data of the Marine Natural Products reviews have also contributed to the free data-sharing platform CMNPD (Comprehensive Marine Natural Products Database)^83^. Those data sources for MNPs provide updated details of bioactive compounds found in taxa from deep-sea habitats to enable the first step in a global, interdisciplinary synthesis.

This study provides a global analysis of bioactivity and new chemical structures in MNPs from deep-sea organisms, characterising the geographic distribution of deep-sea MNPs and the diversity of their structures, bioactivities, and phylogenetic sources. These aspects of deep-sea MNPs could potentially reveal relationships of environmental, physiological and genetic factors with chemical structures and/or bioactivity, whilst also generating a comprehensive gap analysis of biodiscovery in the deep sea. The benefit of including both bioactive and new chemical structures allows for a better understanding of the potential value of these deep-sea environments, since bioactivity alone is narrowly focused by non-standardised assay selection^80^.

An important facet of MNP research is quantifying the potential opportunity cost of anthropogenic activities that may impact these ecosystems and their biodiversity. This study, associated database and interdisciplinary analysis approach may therefore help inform a more targeted approach for future biodiscovery, specifically focusing the screening phase towards specific environments, genetic groups, or data gaps. Additionally, it demonstrates the need for consistent and detailed ecological reporting into a well-maintained database to enable interdisciplinary meta-analyses of bioactivity. This is particularly important in the context of the newly adopted UN Biological Diversity of Areas Beyond National Jurisdiction (BBNJ) agreement (2023)^84^, and we highlight the value of comprehensive MNP-focused databases, namely MarinLit^4^ and CMNPD^83^, which have significantly contributed to the data presented and analysed in this study.

## Results

Our compilation of global data shows 2909 records of compounds and extracts from deep-sea organisms that exhibit either a new chemical structure at the time of publication and/or a positive bioactivity result, i.e., a positive half-maximal inhibitory concentration (IC50) or minimum inhibitory concentration (MIC), irrespective of strength (which we refer to as MNPs). In those 2909 records, 2879 are identified compounds from deep-sea organisms, and the remaining 30 records are extracts whose constituents have not been isolated. Of the 2879 deep-sea compounds catalogued, 34.1% were only structurally “new” at the time of publication, 23.4% only bioactive and the remaining 42.5% demonstrated both bioactivity and new structural chemistry.

**Fig. 1:**
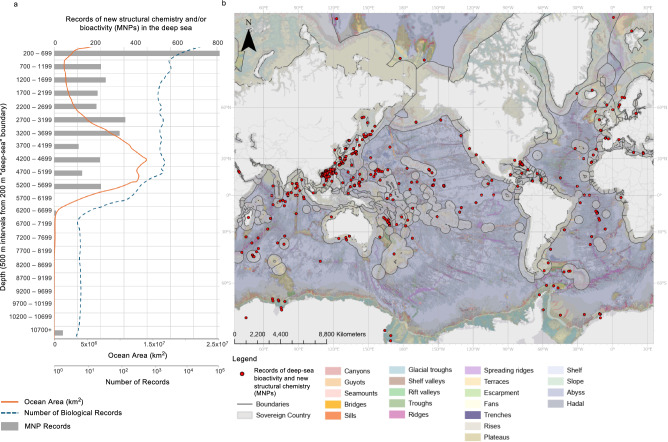
Global distribution of published records of Marine Natural Products (MNPs) from deep-sea organisms: (**a**) distribution of records by depth, compared with ocean area [ref Weatherall et al., 2014]^8^ and number of records [ref Webb et al., 2010]^13^; (**b**) territorial distribution, in relation to Exclusive Economic Zones (EEZs) of nations in the ocean, and geomorphological features according to the Esri hosted 2014 GRID-Arendal map [ref Harris, 2014]^105^. This map was created under licence using ArcGIS Online (Esri, Redlands, CA, USA; https://www.arcgis.com; Accessed June 2025.

Figure 1 shows the geographic, depth, and geomorphological distribution of MNPs isolated from the deep sea. Only 113 entries (3.8%) of the 2909 records in our compiled database did not reference any coordinates for sampling location.

Records of deep-sea MNPs are heavily concentrated in the first 200 to 700 m of water depth (Fig. 1a). From 700 to 5700 m there is a lower sampling effort, which drops to very sparse levels between 6200 m and the hadal depths of the Mariana Trench, where all samples below 7200 m are located. The overall depth distribution of deep-sea MNPs is not representative of the depth distribution of the ocean, for example despite 50% of the earth’s surface lying 3200 m below sea level, only 33% of deep-sea MNPs are derived from collections > 3200 m^8^.

Our data show that 36% of recorded deep-sea MNPs come from samples collected in “High Seas” areas (Fig. 1b) that will be under future jurisdiction of the BBNJ Treaty (BBNJ, 2023) governing Marine Genetic Resources (MGRs). Deep-sea records from High Seas regions represent ~ 2.6% of all MNPs. Within the 64% of deep-sea MNPs collected from EEZs, the majority (40% of all deep-sea MNPs) come from EEZs in the North Pacific (Paracel Islands: 9.9%; Japan: 5.8%; Taiwan 5.4%; New Caledonia: 4.9%; Philippines 4.0%; China: 3.9%; Micronesia: 3.8%), showing an imbalance in MGR exploration between geographic regions.

Only 9% of records are from specimens collected from the water column, with the remaining 91% of records derived from organisms on or in the seabed. Approximately a quarter (27%) of records were sampled from geomorphological seafloor features such as seamounts, guyots, canyons, ridges, troughs, and trenches (Fig. 1b). Some records are concentrated around particular features such as the Mid-Atlantic Ridge, Southwest and Central Indian Ridges, and the Juan de Fuca Ridge in the NE Pacific. Approximately 3% (89 records) of MNPs to date have been identified from deep-sea hydrothermal vents and their proximate sediments, which occur on such seafloor-spreading centres.

### Diversity of phylogeny, structure, and bioactivity in deep-sea MNPs

**Fig. 2:**
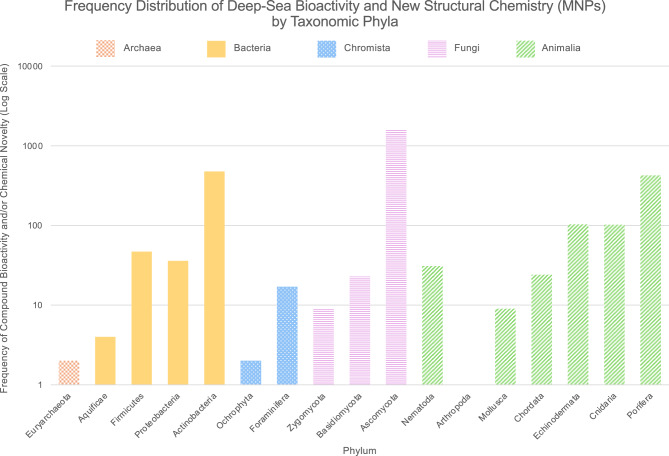
Taxonomic distributions of 2909 published records of Marine Natural Products (MNPs) from deep-sea organisms, showing abundances of deep-sea MNPs by phylum across 4 Kingdoms.

Non-metazoans (Kingdoms Archaea, Fungi, Bacteria, and Chromista) account for ~ 76% of deep-sea MNPs (Fig. 2), with Animalia representing the remaining ~ 24% of records. Within those five Kingdoms, records of deep-sea MNPs occur in 17 phyla with an uneven distribution among them (Fig. 2). Ascomycota fungi represent over half (~ 55%) of deep-sea MNP records to date, and the next most abundant phyla for records of deep-sea MNPs are Actinobacteria (~ 16%) and Porifera (~ 14%).

**Fig. 3:**
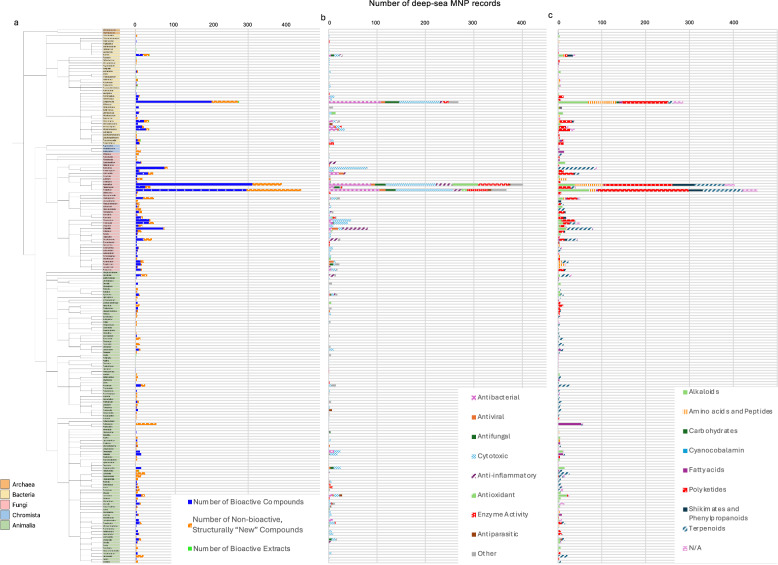
Taxonomic distributions of the characteristics of 2909 published records of Marine Natural Products (MNPs) from deep-sea organisms, generated using iTOL v6.9.1 [ref Letunic & Bork, 2024]^107^: (**a**) abundances of bioactive compounds, new structures, and bioactive extracts by genus; (**b**) abundances of structures of deep-sea MNPs by genus; (**c**) abundances of bioactivities of deep-sea MNPs by genus.

The records within the phyla Ascomycota and Actinobacteria are dominated by the genera *Penicillium* (~ 16% of all deep-sea MNPs) and *Aspergillus* (~ 14%), and *Streptomyces* (~ 10%) respectively (Fig. 3a). Although Animalia contributes 24% of deep-sea MNPs overall, their records are distributed more widely and evenly among genera than in Fungi and Bacteria, with 42 genera contributing to the Porifera records (Fig. 3a).

In terms of structural diversity, polyketides are the most prevalent metabolites in the records of deep-sea MNPs (~ 32%), represented across all Kingdoms (Fig. 3b). Alkaloids, amino acids, peptides, shikimates and phenylpropanoids occur in MNPs from deep-sea Bacteria and Fungi, while Animalia show more fatty acid and terpenoid records (Fig. 3b). This study identifies 1900 extracts and compounds exhibiting single or multiple bioactive properties, amounting to 2290 total recorded bioactive results. The properties most commonly tested for and reported in deep-sea samples are cytotoxic (37%), antibacterial (22%), anti-inflammatory (9%) and enzymatic (8%) activities (Fig. 3c). The distribution of bioactive properties shows that antibacterial compounds are particularly prevalent in *Aspergillus* and *Penicillium* Fungi (Fig. 3c), which have similar profiles of structural diversity and bioactivity. There is also a concentration of enzymatic bioactivity in MNPs from deep-sea *Aspergillus*, while cytotoxic bioactivity is found more evenly across phylogenetic groups (Fig. 3c).

**Fig. 4:**
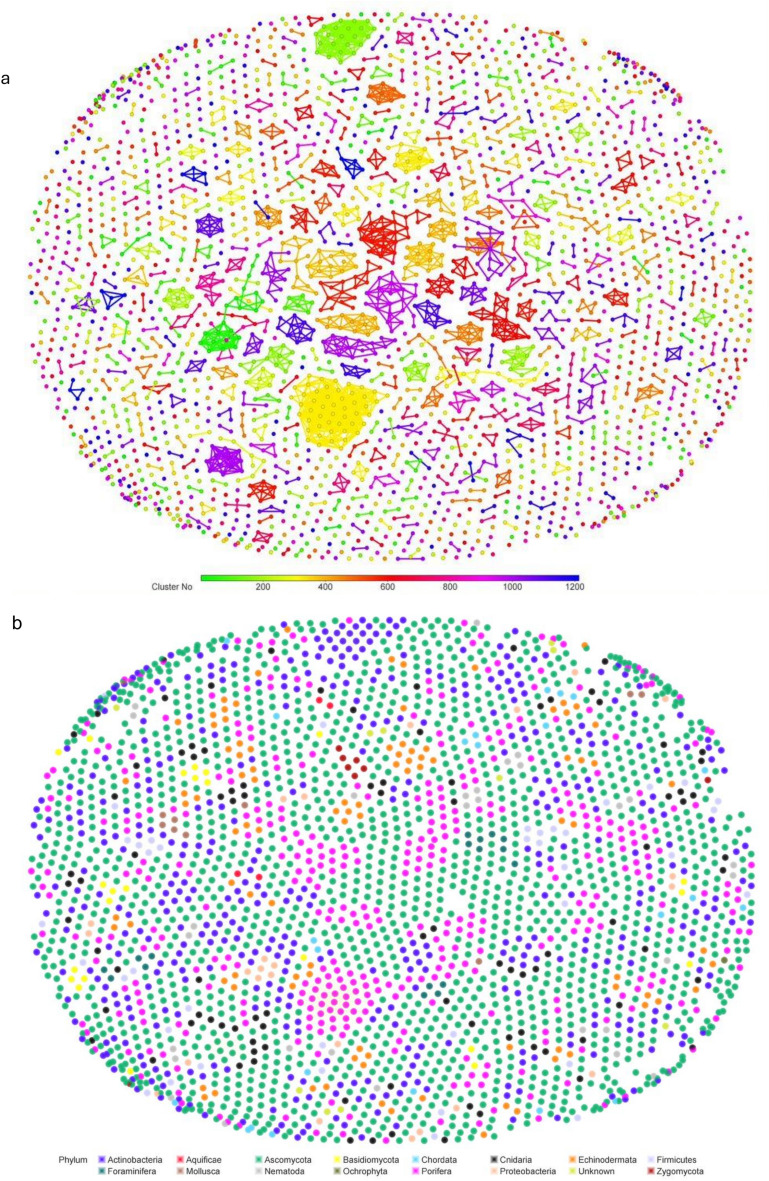
Cluster plot of deep-sea organisms recorded as sources of structurally new and/or bioactive compounds, generated using DataWarrior v06.02.01 [ref Sander et al., 2015]^108^ using a skelspheres descriptor with a 90% similarity index. Links and positioning in (**a**) show similarity of structural scaffolds; (**b**) displays associated phyla.

A cluster analysis of structural similarity (utilising a 90% Tanimoto similarity threshold) among the 2774 records containing deep-sea MNP chemical structures reveals a high prevalence of singletons indicating limited similarity relative to other structures within the dataset Fig. 4a). Some linked clusters correspond to records within individual phyla (Fig. 4b), such as Actinobacteria, Zygomycota, Foraminifera, Porifera, and Echinodermata, indicating possible relationships between phylogeny and the structural chemistry of deep-sea MNPs. The isolation of most points even in more data-abundant phyla such as Ascomycota, however, suggests that there is still a high degree of structural variability even within such phylogenetic groups.

**Fig. 5:**
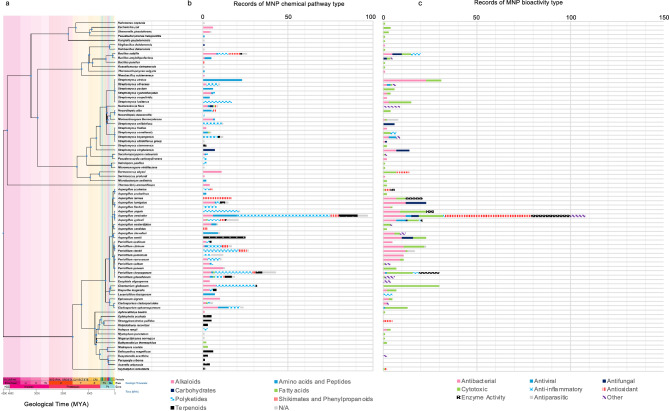
Phylogenetic relationship of 80 deep-sea organisms for which genetic data are available that are sources of Marine Natural Products (MNPs): (**a**) phylogeny of species in relation to geological time generated by TimeTree [Kumar, S. et al*.*, 2022]^103^; (**b**) chemical pathways of MNPs for each species; (**c**) bioactivities of MNPs for each species.

The sources of deep-sea MNPs have been identified to species level in 1711 of records (59%) consisting of 224 species, of which only 80 species have available genetic data (Fig. 5a). Deep-sea MNPs from sources that genetic data are the products of eight biochemical pathways (Fig. 5b) and exhibit eight main types of bioactivity (Fig. 5c), of which cytotoxic (35%) and antibacterial (28%) properties are the most commonly tested and exhibited properties. There appears to be a cluster of antibacterial bioactivity records among closely related *Aspergillus* and *Penicillium* species.

**Fig. 6:**
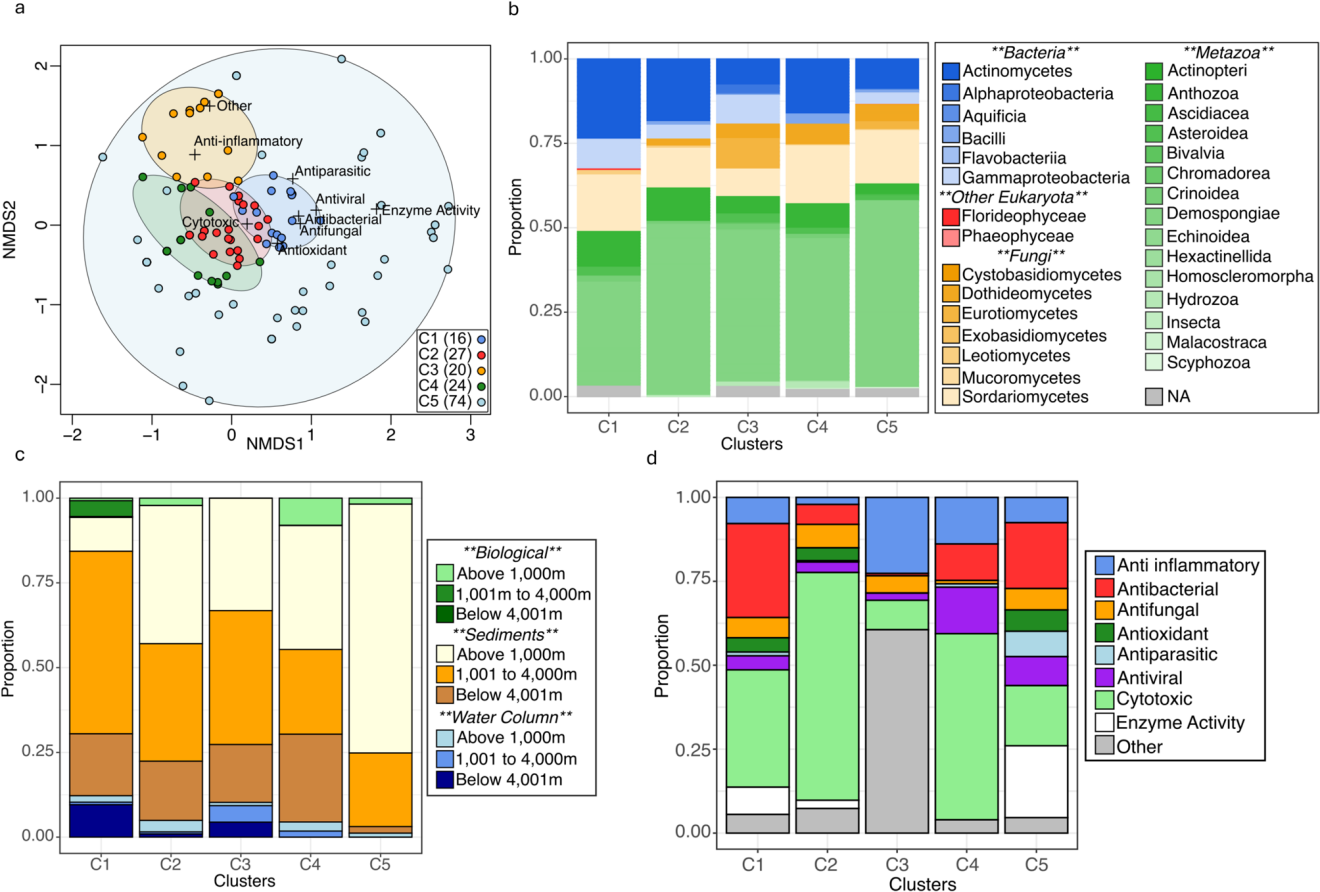
Variation in bioactivity profiles of deep-sea Marine Natural Products: (**a**) non-metric multidimensional scaling (nMDS) combined with K-means cluster analysis for bioactivity profiles, identifying five clusters (C1-5) in the dataset; (**b**) composition of clusters by phylogenetic source; (**c**) composition of clusters by environmental source; (**d**) composition of clusters by activity type.

Cluster analysis of bioactivities reported in deep-sea MNPs identifies five clusters of varying sizes (from 16 to 74 compounds) in the dataset, with a high level of within-cluster variation (Fig. 6a). Phylogenetic and environmental provenance does not vary significantly between clusters (Fig. 6b and c), and biological activity profiles also show no clear trend across clusters (Fig. 6d).

## Discussion

Contrasting environments can exert unusual selective pressures on organisms that may generate new bioactive compounds. Deep-sea environments have been highlighted as a promising frontier for drug discovery as a consequence of heterogeneity in conditions such as temperature, pressure and chemistry^41^. Our study shows that the deep sea remains largely unexplored as a potential reservoir of new, diverse chemical structures and bioactivity, identifying 2909 deep-sea MNPs from published sources and databases. This inventory represents ~ 7.3% of all MNPs^4^, despite the deep sea constituting 90% of global ocean area^8^ and likely containing at least half of estimated marine biodiversity^9^.

The geographic distribution of deep-sea MNPs (Fig. 1a) indicates a sampling bias towards benthic and shallower habitats, similar to the pattern found in records of marine biodiversity^13,17^. Our compilation of data also indicates relatively fewer deep-sea MNP records in areas of the ocean beyond boundaries of national jurisdictions (BBNJ areas), and a high contribution of records from Exclusive Economic Zones (EEZs) in regions such as the North Pacific. Across areas beyond national jurisdiction and EEZs, many deep-sea MNPs also come from sampling at particular geomorphological features that have been the focus of wider marine scientific interest, such as trenches, seamounts and hydrothermal vents.

The geographic distribution of deep-sea MNPs at present therefore reflects sampling effort, rather than possible associations of bioactivities or new structural chemistry with particular deep-sea environments (Fig. 1a). There are 91 records of MNPs from deep-sea hydrothermal vents and their proximate sediments, where organisms thrive in conditions that include mineral-rich hydrothermal fluids, high temperature variation, and in situ chemosynthetic primary production by bacteria and archaea^85^. But deep-sea exploration has been biased towards hydrothermal vents^17^, which may therefore account for the prevalence of deep-sea MNPs from those environments. The idea that the evolution of some MNPs may be related to specific deep-sea niches therefore remains an open question, with a potential for targeted biodiscovery. Perhaps the most extraordinary feature of the deep sea is the extent of the bathymetric environmental gradient that defines it^86^; future research on MNPs should be focussed on sampling across this gradient rather than from particular geomorphological features.

The phylogenetic distribution of deep-sea MNPs is not representative of the full taxonomic diversity known from deep-sea habitats and is likely biased toward taxa that are more easily collected (e.g. sessile metazoa such as sponges), readily cultured, or historically associated with specific bioactivities. Notably, *Aspergillus*, *Penicillium*, and *Streptomyces* together account for 39% of entries in this database, despite likely representing only a small fraction of the total microbial diversity present in deep-sea environments^24,87^.

It is important to note that currently, most MNPs derived from microorganisms are cultured under standard, non-extreme conditions. Further, no new MNPs have been derived from microbes cultured under high pressure, due to extreme condition cultivation challenges, potentially representing a gap in our understanding of MNPs in ~ 76% of this database^18,88^. Furthermore, only 59% of deep-sea MNP records are identified to species level and we have genetic data for only 80 of the 223 species identified. Records of some deep-sea MNPs also lack information about sampling location (3.8%) or chemical structures (4.6%). These gaps in deep-sea MNP data illustrate the need for accurate and detailed reporting in biodiscovery to enable interdisciplinary meta-analyses^89^.

Phylogeographic analysis could aid biodiscovery through better understanding of the genetic divergence of species inhabiting certain deep-sea conditions and the implications for their molecular chemistry. Deep-sea ctenophores, for example, exhibit adapted phospholipids that enhance pressure tolerance in *Escherichia coli*^90^. Our analysis, however, shows that more data are required before we can establish relationships, if any, between phylogeny and bioactivity, and certainly before phylogenetically targeted biodiscovery can be developed in the deep sea. At present, the absence of a chemical pathway or bioactivity within a phylogenetic group is likely to result from limited sampling. It is also further confounded by the tendency not to publish or record negative results, whereby absence of evidence cannot be interpreted as evidence of absence.

Our analysis shows the diversity of compound structures within current records of deep-sea MNPs. Whereby, using skelsphere descriptors, few structures demonstrate similarity indices of more than 90% (Fig. 4a), which is potentially an indication of a reservoir of unique chemistry. This is not however, an indicator that deep-sea chemistry is more or less diverse or unique compared with shallow water or terrestrial environments. Whilst previous studies have demonstrated that the deep sea represents a unique reservoir of novel chemistry, it has also been shown that 76.7% of microorganism-derived MNPs are closely related to compounds isolated from terrestrial microorganisms^18,43^. This pattern may partly reflect: the collection of resistant spores transported from land to the ocean that can remain viable but dormant for years^91^, that microroganisms capable of being cultured within current culture media biases and non-extreme cultivation conditions express similar chemical structures to terrestrial or shallow water micororganisms or that the use of conserved biosynthetic resources and chemical building blocks are widely shared across deep-sea and terrestrial microorganisms^18,88,92^. Thus, further study, particularly of macro-organisms, could reveal the comparative uniqueness of deep-sea MNPs by conducting a similar structural skelspheres analysis that includes a dataset of all known shallow water and terrestrial natural product chemistry. Additionally, Fig. 4b shows some linkage of similar structures with particular phylogenetic groups. The high degree of structural variability within groups found in our analysis, however, highlights the potential for more complete databases to detect meaningful trends.

Our analysis of deep-sea MNP data highlight the current trends and gaps in deep-sea biodiscovery, and the need for comprehensive records for bioactive or structurally new MNPs. Further, it is noteworthy, that our analysis of MNPs is limited to the quality of chemical structure and bioactivity reporting, much of which is intrinsically biased towards a known chemical space, e.g., towards research group preference and taxa that have already shown positive results^93,94^, and include bioactivity testing that is often not effectively standardised^80^. It is important that accurate and detailed reporting, including genetic information, becomes a more regular part of the biodiscovery process, and that data should be recorded in accordance with FAIR (Findable, Accessible, Interoperable, Reusable) principles included in Article 14 paragraph 2(c) of the BBNJ Agreement^84,95^, for example using open access databases such as OBIS (2024) (https://obis.org/)^96^. Data sourced from areas beyond national jurisdiction will also need to conform with the Clearing-House Mechanism prescribed by the Article 51 of the BBNJ Agreement, which entered into force on 17 January 2026^84^.

Overall, these data recording enhancements may enable a better understanding of the phylogenetic distribution of bioactivity/new chemical structures in deep-sea MNPs, and environmental correlations in compound expression, leading to future phylogeographic analysis and computational pharmaceutical screening that could aid targeted biodiscovery.

A systematic survey of MNPs across deep-sea environments and taxa, including recording absences of bioactivities and new sources of already-known compounds, could substantially boost such an effort, similar to the ambition of the international Ocean Census, to advance knowledge of deep-sea biodiversity as part of the UN Ocean Decade^97^. Future comparative analysis of deep-sea MNPs with compounds from shallow-water and terrestrial sources would be beneficial to understand the uniqueness of deep-sea chemistry^92^. Improving our understanding of the deep sea as a source of MNPs is urgently required to inform the evaluation of activities such as deep-sea mining, which may have impacts on genetic diversity in deep-sea environments^98^.

## Methods

### Compilation of deep-sea MNP data

The data presented and analysed in this study were compiled from a thorough independent literature review, which utilised key words such as, “deep sea”, “bioactivity”, “chemistry”, “compounds”, “novel”, “new”, “scaffolds”, “structures”, “IC50”, “MIC” and all the bioactivity categories listed in Fig. 3c within academic search platforms including Google Scholar and PubMed. The commercial database MarinLit (https://marinlit.rsc.org/)^4^ and the free data sharing platform, CMNPD (https://www.cmnpd.org) ^83^, were valuable resources in signposting relevant literature, which were filtered and cross-checked to only include records below 200 m. Details of every entry can be found in the supplementary data. To qualify as an MNP in this dataset, each record had to be derived from a biological source > 200 m deep. The chemical structure at the time of publication had to be regarded as “new” or “novel” and/or generate a positive bioactivity result, i.e., a positive half-maximal inhibitory concentration (IC50) or minimum inhibitory concentration (MIC), irrespective of strength.

The data were cross-checked and developed in more detail using the open-access and open-source platforms Reaxys (https://www.reaxys.com/)^99^, Chemspider (https://www.chemspider.com/)^100^, Natural Product (NP) Classifier (https://npclassifier.ucsd.edu/)^101^, World Register of Marine Species (WoRMS) (https://www.marinespecies.org/)^102^ and TimeTree (https://timetree.org/)^103^ to build a database of chemical structures, chemical classifications, taxonomy and phylogeny respectively for each MNP record. Table 1 of the supplementary material provides a summary of the information acquired for each record where possible. Table 2 of the supplementary material provides a general, chemical, taxonomic and bioactivity summary of the curated database.

### Visualisation and analysis of phylogenetic, structural and bioactivity data

Geographic distribution data (Fig. 1b) were plotted using ARCGIS Pro 3.5.0^104^, and analysed against the GRID Arendal World Seafloor Geomorphology^105^ and Economic Zone map image layers^106^. Taxonomic plots (Fig. 3) were created using version 6.9.1. of the “Interactive Tree of Life” platform (iTOL v6.9.1)^107^. Records that could not be identified to genus level were excluded from the plot and the remaining genera were plotted in terms of taxonomic classifications defined by WoRMS^102^. The taxonomic trees of genera were manually coded using PhyloXML formatting and imported into the iTOL v6.9.1 programme as a txt. file. Version 1.8 of the “iTOL annotation editor”^107^ was used for formatting and to plot bar charts against the taxonomic trees.

Cluster analysis of chemical structures (Fig. 4) was conducted using DataWarrior v06.02.01^108^, using a skelspheres descriptor with a 90% similarity index. The 90% Tanimoto similarity threshold was intentionally selected to identify and visualise closely related structural scaffolds among deep-sea–derived bioactive compounds. At this high threshold, clustering emphasises fine-grained scaffold conservation, enabling the detection of subtle structural variations. To highlight the influence of Tanimoto similarity threshold selection, Fig. 1 of the supplementary material offers an 80% similarity index. Figure 5a utilised the TimeTree platform^103^ to plot the phylogeny of species against geological time. 80 species within the database had sufficient genetic data to be recognised by the TimeTree platform and included in the analysis to visualise any relationships between phylogenetics (Fig. 5a) and chemical pathway and bioactivity type (Fig. 5b and c). Non-metric multidimensional scaling (nMDS) with K-means cluster analysis on Bray–Curtis dissimilarities was used to explore similarity in biological activity profiles across compounds (Fig. 6a). Due to the high level of group dispersion as well as uneven group sizes, PERMANOVA analysis was not performed^109^. Instead, cluster number was selected by minimising gap statistics, a measure which compares the within cluster dispersion against an expected null distribution^110^. Taxonomic classification, environmental provenance and biological activity profiles of compounds in each cluster were then compared (Fig. 6b, c and d).

## Supplementary Information

Supplementary Information 1.

Supplementary Information 2.

## Data Availability

All relevant data and information have been provided in the supplementary data and supplementary material files.

## References

[1] Newman, D. J. & Cragg, G. M. Natural products as sources of new drugs over the nearly four decades from 01/1981 to 09/2019. *J. Nat. Prod.* **83**, 770–803. 10.1021/acs.jnatprod.9b01285 (2020). 32162523 10.1021/acs.jnatprod.9b01285

[2] WHO. Ageing and health, <https://www.who.int/news-room/fact-sheets/detail/ageing-and-health> (2024).

[3] Ventola, C. L. The antibiotic resistance crisis: Part 1: Causes and threats. *Pharm. Ther.* **40**, 277–283 (2015). PMC4378521 25859123

[4] Royal society of chemistry. MarinLit. https://marinlit.rsc.org Accessed July 2025 (2024).

[5] Francesch, A. et al. Pharmaceuticals from marine sources: past, present and future pharmaceutical-journal.com/article/research/pharmaceuticals-from-marine-sources-past-present-and-future. *Pharm. J.*10.1211/PJ.2024.1.324083 (2024).

[6] Kong, D.-X., Jiang, Y.-Y. & Zhang, H.-Y. Marine natural products as sources of novel scaffolds: Achievement and concern. *Drug Discov. Today* **15**, 884–886. 10.1016/j.drudis.2010.09.002 (2010). 20869461 10.1016/j.drudis.2010.09.002

[7] Spainhour, C. B. Drug Discovery Handbook (ed S.C. Gad) Ch. 1, 11–72 (2005).

[8] Weatherall, P. et al. A new digital bathymetric model of the world’s oceans. *Earth Space Sci.* **2**, 331–345. 10.1002/2015EA000107 (2015).

[9] Mora, C., Tittensor, D. P., Adl, S., Simpson, A. G. B. & Worm, B. How many species are there on Earth and in the ocean?. *PLoS Biol.* **9**, e1001127. 10.1371/journal.pbio.1001127 (2011). 21886479 10.1371/journal.pbio.1001127 PMC3160336

[10] Merino, N. et al. Living at the extremes: extremophiles and the limits of life in a planetary context. *Front. Microbiol.*10.3389/fmicb.2019.00780 (2019). 31037068 10.3389/fmicb.2019.00780 PMC6476344

[11] Paulus, E. Shedding light on deep-sea biodiversity—A highly vulnerable habitat in the face of anthropogenic change.. *Front. Mar. Sci.*10.3389/fmars.2021.667048 (2021).

[12] Arico, S. & Salpin, C. Bioprospecting of Genetic Resources in the Deep Seabed: Scientific, Legal and Policy Aspects. 10, 28 (The Convention on Biological Diversity, United Nations University Institute of Advanced Studies, (2005).

[13] Webb, T. J., Vanden Berghe, E. & O’Dor, R. Biodiversity’s big wet secret: The global distribution of marine biological records reveals chronic under-exploration of the deep pelagic ocean. *PLoS ONE* **5**, e10223. 10.1371/journal.pone.0010223 (2010). 20689845 10.1371/journal.pone.0010223 PMC2914017

[14] Wright, P. C., Westacott, R. E. & Burja, A. M. Piezotolerance as a metabolic engineering tool for the biosynthesis of natural products. *Biomol. Eng.* **20**, 325–331. 10.1016/S1389-0344(03)00042-X (2003). 12919816 10.1016/s1389-0344(03)00042-x

[15] Bull, A. T., Ward, A. C. & Goodfellow, M. Search and discovery strategies for biotechnology: The paradigm shift.. *Microbiol. Mol. Biol. Rev.* **64**, 573–606 (2000). 10974127 10.1128/mmbr.64.3.573-606.2000 PMC99005

[16] Danovaro, R. et al. Implementing and innovating marine monitoring approaches for assessing marine environmental status. *Front. Marine Sci.*10.3389/fmars.2016.00213 (2016).

[17] Bell, K. L. C., Johannes, K. N., Kennedy, B. R. C. & Poulton, S. E. How little we’ve seen: A visual coverage estimate of the deep seafloor.. *Sci. Adv.* **11**, eadp8602 (2025). 40333982 10.1126/sciadv.adp8602 PMC12057672

[18] Khan, S., Wang, T., Arifeen, M. Z. U. & Huang, S. Exploring the bioactive potential of deep-sea microorganisms: A review of recent discoveries. *Bioorg. Chem.*10.1016/j.bioorg.2025.108521 (2025). 40373561 10.1016/j.bioorg.2025.108521

[19] Boucher, G. & Lambshead, P. J. D. Ecological biodiversity of marine nematodes in samples from temperate, tropical, and deep-sea regions. *Conserv. Biol.* **9**, 1594–1604 (1995).

[20] Glover, A. G. & Smith, C. R. The deep-sea floor ecosystem: Current status and prospects of anthropogenic change by the year 2025. *Environ. Conserv.* **30**, 219–241 (2003).

[21] Grassle, J. F., Maciolek, N. J. & Blake, J. A. 19 are deep-sea communities resilient?.

[22] McClain, C. R. & Schlacher, T. A. On some hypotheses of diversity of animal life at great depths on the sea floor. *Mar. Ecol.* **36**, 849–872 (2015).

[23] Zhang, C., Peng, Y., Liu, X., Wang, J. & Dong, X. Deep-sea microbial genetic resources: New frontiers for bioprospecting. *Trends Microbiol.* **32**, 321–324. 10.1016/j.tim.2024.01.002 (2024). 38290879 10.1016/j.tim.2024.01.002

[24] Sogin, M. L. et al. Microbial diversity in the deep sea and the underexplored “rare biosphere”. *Proc. Natl. Acad. Sci. U. S. A.* **103**, 12115–12120. 10.1073/pnas.0605127103 (2006). 16880384 10.1073/pnas.0605127103 PMC1524930

[25] Bridges, A. E. H. & Howell, K. L. Prioritisation of ocean biodiversity data collection to deliver a sustainable ocean. *Commun. Earth Environ.* **6**, 473. 10.1038/s43247-025-02442-7 (2025). 40546268 10.1038/s43247-025-02442-7 PMC12176621

[26] Capon, R. J. Marine bioprospecting − Trawling for treasure and pleasure. *Eur. J. Org. Chem.* **2001**, 633–645. 10.1002/1099-0690(200102)2001:4<633::AID-EJOC633>3.0.CO;2-Q (2001).

[27] Winder, P. L., Pomponi, S. A. & Wright, A. E. Natural products from the Lithistida: A review of the literature since 2000. *Mar. Drugs* **9**, 2643–2682. 10.3390/md9122643 (2011). 22363244 10.3390/md9122643 PMC3280575

[28] Bewley, C. A. & Faulkner, D. J. Lithistid sponges: Star performers or hosts to the stars. *Angew. Chem. Int. Ed.* **37**, 2162–2178. 10.1002/(SICI)1521-3773(19980904)37:16<2162::AID-ANIE2162>3.0.CO;2-2 (1998). 10.1002/(SICI)1521-3773(19980904)37:16<2162::AID-ANIE2162>3.0.CO;2-2 29711453

[29] Thomas, T. R., Kavlekar, D. P. & LokaBharathi, P. A. Marine drugs from sponge-microbe association--A review. *Mar. Drugs* **8**, 1417–1468. 10.3390/md8041417 (2010). 20479984 10.3390/md8041417 PMC2866492

[30] Wang, Y. T., Xue, Y. R. & Liu, C. H. A brief review of bioactive metabolites derived from deep-sea fungi. *Mar. Drugs* **13**, 4594–4616. 10.3390/md13084594 (2015). 26213949 10.3390/md13084594 PMC4556995

[31] Wang, Z. et al. Progress in the discovery of new bioactive substances from deep-sea associated fungi during 2020-2022. *Front. Mar. Sci.*10.3389/fmars.2023.1232891 (2023).

[32] Kamjam, M., Sivalingam, P., Deng, Z. & Hong, K. Deep sea actinomycetes and their secondary metabolites. *Front. Microbiol.*10.3389/fmicb.2017.00760 (2017). 28507537 10.3389/fmicb.2017.00760 PMC5410581

[33] Pettit, R. K. Culturability and secondary metabolite diversity of extreme microbes: Expanding contribution of deep sea and deep-sea vent microbes to natural product discovery. *Mar. Biotechnol. (NY)* **13**, 1–11. 10.1007/s10126-010-9294-y (2011). 20437069 10.1007/s10126-010-9294-y

[34] Wang, Y. N., Meng, L. H. & Wang, B. G. Progress in research on bioactive secondary metabolites from deep-sea derived microorganisms. *Mar. Drugs*10.3390/md18120614 (2020). 33276592 10.3390/md18120614 PMC7761599

[35] Wilson, Z. E. & Brimble, M. A. Molecules derived from the extremes of life: A decade later. *Nat. Prod. Rep.* **38**, 24–82. 10.1039/D0NP00021C (2021). 32672280 10.1039/d0np00021c

[36] Thornburg, C. C., Zabriskie, T. M. & McPhail, K. L. Deep-sea hydrothermal vents: Potential hot spots for natural products discovery?. *J. Nat. Prod.* **73**, 489–499. 10.1021/np900662k (2010). 20099811 10.1021/np900662k

[37] Laurent, D. & Pietra, F. Natural-product diversity of the New Caledonian marine ecosystem compared to other ecosystems: A pharmacologically oriented view. *Chem. Biodivers.* **1**, 539–594. 10.1002/cbdv.200490048 (2004). 17191868 10.1002/cbdv.200490048

[38] Lebar, M. D., Heimbegner, J. L. & Baker, B. J. Cold-water marine natural products. *Nat. Prod. Rep.* **24**, 774–797. 10.1039/B516240H (2007). 17653359 10.1039/b516240h

[39] Russo, P., Del Bufalo, A. & Fini, M. Deep sea as a source of novel-anticancer drugs: Update on discovery and preclinical/clinical evaluation in a systems medicine perspective. *EXCLI J.* **14**, 228–236. 10.17179/excli2015-632 (2015). 26600744 10.17179/excli2014-632 PMC4652633

[40] Tortorella, E. et al. Antibiotics from deep-sea microorganisms: Current discoveries and perspectives. *Mar. Drugs*10.3390/md16100355 (2018). 30274274 10.3390/md16100355 PMC6213577

[41] Skropeta, D. Deep-sea natural products. *Nat. Prod. Rep.* **25**, 1131–1166. 10.1039/b808743a (2008). 19030606 10.1039/b808743a

[42] Skropeta, D. & Wei, L. Recent advances in deep-sea natural products. *Nat. Prod. Rep.* **31**, 999–1025. 10.1039/C3NP70118B (2014). 24871201 10.1039/c3np70118b

[43] Saide, A., Lauritano, C. & Ianora, A. A treasure of bioactive compounds from the deep sea. *Biomedicines* **9**, 1556 (2021). 34829785 10.3390/biomedicines9111556 PMC8614969

[44] Faulkner, D. J. Marine natural products. *Nat. Prod. Rep.* **3**, 1–33. 10.1039/NP9860300001 (1986). 2872636 10.1039/np9860300001

[45] Faulkner, D. J. Marine natural products. *Nat. Prod. Rep.* **4**, 539–576. 10.1039/NP9870400539 (1987). 3328831 10.1039/np9870400539

[46] Faulkner, D. J. Marine natural products. *Nat. Prod. Rep.* **5**, 613–663. 10.1039/NP9880500613 (1988). 3072499 10.1039/np9880500613

[47] Faulkner, D. J. Marine natural products. *Nat. Prod. Rep.* **7**, 269–309. 10.1039/NP9900700269 (1990). 2250800 10.1039/np9900700269

[48] Faulkner, D. J. Marine natural products. *Nat. Prod. Rep.* **8**, 97–147. 10.1039/NP9910800097 (1991). 1679913 10.1039/np9910800097

[49] Faulkner, D. J. Marine natural products. *Nat. Prod. Rep.* **9**, 323–364. 10.1039/NP9920900323 (1992).

[50] Faulkner, D. J. Marine natural products. *Nat. Prod. Rep.* **10**, 497–539. 10.1039/np9931000497 (1993). 8295744 10.1039/np9931000497

[51] Faulkner, D. J. Marine natural products.. *Nat. Prod. Rep.* **11**, 355–394. 10.1039/np9941100355 (1994). 15200019 10.1039/np9941100355

[52] Faulkner, D. J. Marine natural products.. *Nat. Prod. Rep.* **12**, 223–269. 10.1039/NP9951200223 (1995).

[53] Faulkner, D. J. Marine natural products.. *Nat. Prod. Rep.* **13**, 75–125. 10.1039/NP9961300075 (1996). 8622813 10.1039/np9961300075

[54] Faulkner, D. J. Marine natural products.. *Nat. Prod. Rep.* **14**, 259–302. 10.1039/NP9971400259 (1997).

[55] John Faulkner, D. Marine natural products. *Nat. Prod. Rep.* **15**, 113–158. 10.1039/A815113Y (1998). 9586223 10.1039/a815113y

[56] John Faulkner, D. Marine natural products. *Nat. Prod. Rep.* **16**, 155–198. 10.1039/A804469D (1999).

[57] John Faulkner, D. Marine natural products. *Nat. Prod. Rep.* **17**, 7–55. 10.1039/A809395D (2000). 10714898 10.1039/a809395d

[58] Faulkner, D. J. Marine natural products.. *Nat. Prod. Rep.* **18**, 1R-49R. 10.1039/B006897G (2001). 11245399 10.1039/b006897g

[59] Faulkner, D. J. Marine natural products.. *Nat. Prod. Rep.* **19**, 1–49. 10.1039/B009029H (2002). 11902436 10.1039/b009029h

[60] Faulkner, D. J. Marine natural products: Metabolites of marine algae and herbivorous marine molluscs.. *Nat. Prod. Rep.* **1**, 251–280. 10.1039/NP9840100251 (1984).

[61] Faulkner, D. J. Marine natural products: Metabolites of marine invertebrates. *Nat. Prod. Rep.* **1**, 551–598. 10.1039/NP9840100551 (1984).

[62] Blunt, J. W., Copp, B. R., Munro, M. H. G., Northcote, P. T. & Prinsep, M. R. Marine natural products. *Nat. Prod. Rep.* **20**, 1–48. 10.1039/B207130B (2003). 12636082 10.1039/b207130b

[63] Blunt, J. W., Copp, B. R., Munro, M. H. G., Northcote, P. T. & Prinsep, M. R. Marine natural products. *Nat. Prod. Rep.* **21**, 1–49. 10.1039/B305250H (2004). 15039834 10.1039/b305250h

[64] Blunt, J. W., Copp, B. R., Munro, M. H. G., Northcote, P. T. & Prinsep, M. R. Marine natural products. *Nat. Prod. Rep.* **22**, 15–61. 10.1039/B415080P (2005). 15692616 10.1039/b415080p

[65] Blunt, J. W., Copp, B. R., Munro, M. H. G., Northcote, P. T. & Prinsep, M. R. Marine natural products. *Nat. Prod. Rep.* **23**, 26–78. 10.1039/B502792F (2006). 16453031 10.1039/b502792f

[66] Blunt, J. W. et al. Marine natural products. *Nat. Prod. Rep.* **24**, 31–86. 10.1039/B603047P (2007). 17268607 10.1039/b603047p

[67] Blunt, J. W. et al. Marine natural products. *Nat. Prod. Rep.* **25**, 35–94. 10.1039/B701534H (2008). 18250897 10.1039/b701534h

[68] Blunt, J. W. et al. Marine natural products. *Nat. Prod. Rep.* **26**, 170–244. 10.1039/B805113P (2009). 19177222 10.1039/b805113p

[69] Blunt, J. W., Copp, B. R., Munro, M. H. G., Northcote, P. T. & Prinsep, M. R. Marine natural products. *Nat. Prod. Rep.* **27**, 165–237. 10.1039/B906091J (2010). 20111802 10.1039/b906091j

[70] Blunt, J. W., Copp, B. R., Munro, M. H. G., Northcote, P. T. & Prinsep, M. R. Marine natural products. *Nat. Prod. Rep.* **28**, 196–268. 10.1039/C005001F (2011). 21152619 10.1039/c005001f

[71] Blunt, J. W., Copp, B. R., Keyzers, R. A., Munro, M. H. G. & Prinsep, M. R. Marine natural products. *Nat. Prod. Rep.* **29**, 144–222. 10.1039/C2NP00090C (2012). 22193773 10.1039/c2np00090c

[72] Blunt, J. W., Copp, B. R., Keyzers, R. A., Munro, M. H. G. & Prinsep, M. R. Marine natural products. *Nat. Prod. Rep.* **30**, 237–323. 10.1039/C2NP20112G (2013). 23263727 10.1039/c2np20112g

[73] Blunt, J. W., Copp, B. R., Keyzers, R. A., Munro, M. H. G. & Prinsep, M. R. Marine natural products. *Nat. Prod. Rep.* **32**, 116–211. 10.1039/C4NP00144C (2015). 25620233 10.1039/c4np00144c

[74] Blunt, J. W., Copp, B. R., Keyzers, R. A., Munro, M. H. G. & Prinsep, M. R. Marine natural products. *Nat. Prod. Rep.* **33**, 382–431. 10.1039/C5NP00156K (2016). 26837534 10.1039/c5np00156k

[75] Blunt, J. W., Copp, B. R., Keyzers, R. A., Munro, M. H. G. & Prinsep, M. R. Marine natural products. *Nat. Prod. Rep.* **34**, 235–294. 10.1039/C6NP00124F (2017). 28290569 10.1039/c6np00124f

[76] Blunt, J. W. et al. Marine natural products. *Nat. Prod. Rep.* **35**, 8–53. 10.1039/C7NP00052A (2018). 29335692 10.1039/c7np00052a

[77] Carroll, A. R., Copp, B. R., Davis, R. A., Keyzers, R. A. & Prinsep, M. R. Marine natural products. *Nat. Prod. Rep.* **36**, 122–173. 10.1039/C8NP00092A (2019). 30663727 10.1039/c8np00092a

[78] Carroll, A. R., Copp, B. R., Davis, R. A., Keyzers, R. A. & Prinsep, M. R. Marine natural products. *Nat. Prod. Rep.* **37**, 175–223. 10.1039/C9NP00069K (2020). 32025684 10.1039/c9np00069k

[79] Carroll, A. R., Copp, B. R., Davis, R. A., Keyzers, R. A. & Prinsep, M. R. Marine natural products. *Nat. Prod. Rep.* **38**, 362–413. 10.1039/D0NP00089B (2021). 33570537 10.1039/d0np00089b

[80] Carroll, A. R., Copp, B. R., Davis, R. A., Keyzers, R. A. & Prinsep, M. R. Marine natural products. *Nat. Prod. Rep.* **39**, 1122–1171. 10.1039/D1NP00076D (2022). 35201245 10.1039/d1np00076d

[81] Carroll, A. R., Copp, B. R., Davis, R. A., Keyzers, R. A. & Prinsep, M. R. Marine natural products. *Nat. Prod. Rep.* **40**, 275–325. 10.1039/D2NP00083K (2023). 36786022 10.1039/d2np00083k

[82] Carroll, A. R., Copp, B. R., Grkovic, T., Keyzers, R. A. & Prinsep, M. R. Marine natural products. *Nat. Prod. Rep.* **41**, 162–207. 10.1039/D3NP00061C (2024). 38285012 10.1039/d3np00061c

[83] Lyu, C. et al. CMNPD: A comprehensive marine natural products database towards facilitating drug discovery from the ocean. *Nucleic Acids Res.* **49**, D509-d515. 10.1093/nar/gkaa763 (2021). 32986829 10.1093/nar/gkaa763 PMC7779072

[84] Agreement Under the United Nations Convention on the Law of the Sea on the Conservation and Sustainable Use of Marine Biological Diversity of Areas Beyond National Jurisdiction (BBNJ). *BBNJ* (Adopted June 19, 2023).

[85] Andrianasolo, E., Lutz, R. & Falkowski, P. Studies in Natural Products Chemistry Vol. 36 (ed R. Atta ur) Ch. 3, 43–66 (Elsevier, 2012).

[86] Rex, M. A. Community structure in the deep-sea benthos. *Annu. Rev. Ecol. Syst.* **12**, 331–353 (1981).

[87] Yang, S., Xu, W., Gao, Y., Chen, X. & Luo, Z.-H. Fungal diversity in deep-sea sediments from Magellan Seamounts environment of the Western Pacific revealed by high-throughput Illumina sequencing. *J. Microbiol.* **58**, 841–852 (2020). 32876913 10.1007/s12275-020-0198-x

[88] Fulke, A. B., Sharma, N. & Nadekar, J. Darkness to discovery: A comprehensive mini-review on culturable and non-culturable microbial diversity from deep sea. *Microb. Ecol.* **88**, 77 (2025). 40679638 10.1007/s00248-025-02527-y PMC12274225

[89] Leal, M. C., Hilário, A., Munro, M. H., Blunt, J. W. & Calado, R. Natural products discovery needs improved taxonomic and geographic information. *Nat. Prod. Rep.* **33**, 747–750. 10.1039/c5np00130g (2016). 26892141 10.1039/c5np00130g

[90] Winnikoff, J. R. et al. Homeocurvature adaptation of phospholipids to pressure in deep-sea invertebrates. *Science* **384**, 1482–1488. 10.1126/science.adm7607 (2024). 38935710 10.1126/science.adm7607 PMC11593575

[91] Mincer, T. J., Jensen, P. R., Kauffman, C. A. & Fenical, W. Widespread and persistent populations of a major new marine actinomycete taxon in ocean sediments. *Appl. Environ. Microbiol.* **68**, 5005–5011. 10.1128/AEM.68.10.5005-5011.2002 (2002). 12324350 10.1128/AEM.68.10.5005-5011.2002 PMC126404

[92] Voser, T. M., Campbell, M. D. & Carroll, A. R. How different are marine microbial natural products compared to their terrestrial counterparts?. *Nat. Prod. Rep.* **39**, 7–19. 10.1039/D1NP00051A (2022). 34651634 10.1039/d1np00051a

[93] Wee, D. & Chisowa, T. The great unknown: How chemistry remains the last frontier to understanding life. *GEN Biotechnol.* **4**, 114–118 (2025).

[94] Vitale, G. A. et al. Connecting metabolome and phenotype: Recent advances in functional metabolomics tools for the identification of bioactive natural products. *Nat. Prod. Rep.* **41**, 885–904 (2024). 38351834 10.1039/d3np00050h PMC11186733

[95] Wilkinson, M. D. et al. The FAIR guiding principles for scientific data management and stewardship. *Sci. Data* **3**, 160018. 10.1038/sdata.2016.18 (2016). 26978244 10.1038/sdata.2016.18 PMC4792175

[96] OBIS. (Intergovernmental Oceanographic Commission of UNESCO, 2024).

[97] Rogers, A. D. & Ramirez Llodra, E. Deep-sea exploration of marine ecosystems – Knowledge and solutions for marine biodiversity. *Int. Hydrogr. Rev.* **30** (2025).

[98] Niner, H. J. et al. Deep-sea mining with no net loss of biodiversity—An impossible aim. *Front. Mar. Sci.*10.3389/fmars.2018.00053 (2018).

[99] Elsevier. Reaxys, <https://www.reaxys.com/#/search/quick/query> (2024).

[100] Pence, H. E. & Williams, A. ChemSpider: An online chemical information resource. *J. Chem. Educ.* **87**, 1123–1124. 10.1021/ed100697w (2010).

[101] Kim, H. W. et al. NPClassifier: A deep neural network-based structural classification tool for natural products. *J. Nat. Prod.* **84**, 2795–2807. 10.1021/acs.jnatprod.1c00399 (2021). 34662515 10.1021/acs.jnatprod.1c00399 PMC8631337

[102] Ahyong, S. *et al.* (WoRMS Editorial Board, 2025).

[103] Kumar, S. et al. TimeTree 5: An expanded resource for species divergence times. *Mol. Biol. Evol.*10.1093/molbev/msac174 (2022). 35932227 10.1093/molbev/msac174 PMC9400175

[104] ESRI. (Redlands, CA, 2025).

[105] Harris, P. World Seafloor Geomorphology from GRID Arendal (ESRI, 2014).

[106] FMI. (Flanders Marine Institute, 2023).

[107] Letunic, I. & Bork, P. Interactive tree of life (iTOL) v6: Recent updates to the phylogenetic tree display and annotation tool. *Nucleic Acids Res.* **52**, W78-w82. 10.1093/nar/gkae268 (2024). 38613393 10.1093/nar/gkae268 PMC11223838

[108] Sander, T., Freyss, J., von Korff, M. & Rufener, C. DataWarrior: An open-source program for chemistry aware data visualization and analysis. *J. Chem. Inf. Model.* **55**, 460–473. 10.1021/ci500588j (2015). 25558886 10.1021/ci500588j

[109] Anderson, M. J. & Walsh, D. C. I. PERMANOVA, ANOSIM, and the Mantel test in the face of heterogeneous dispersions: What null hypothesis are you testing?. *Ecol. Monogr.* **83**, 557–574. 10.1890/12-2010.1 (2013).

[110] Tibshirani, R., Walther, G. & Hastie, T. Estimating the number of clusters in a data set via the Gap Statistic.. *J. R. Stat. Soc. B* **63**, 411–423 (2001).

