# Supplementary material for: Global distribution of deep-sea natural products shows environmental and phylogenetic undersampling with potential for biodiscovery
Source: Sci Rep. 2026 Jul 10;16:21496. doi: 10.1038/s41598-026-45398-0 (PMC13350919; doi:10.1038/s41598-026-45398-0)

Supplementary Material

Table 1. A description of the data categories used in the curation of an interdisciplinary database of chemical novelty and bioactivity in the deep sea by 2024.

Data Category	Description
Domain	Taxonomic Domain identified by the publication and defined by WoRMs[98] if available.
Kingdom	Taxonomic Kingdom identified by the publication defined by WoRMs[98] if available.
Phylum	Taxonomic Phylum identified by the publication defined by WoRMs[98] if available.
Class	Taxonomic Class identified by the publication defined by WoRMs[98] if available.
Order	Taxonomic Order identified by the publication defined by WoRMs[98] if available.
Family	Taxonomic Family identified by the publication defined by WoRMs[98] if available.
Genus	Taxonomic Genus identified by the publication defined by WoRMs[98] if available.
Species	Taxonomic Species identified by the publication defined by WoRMs[98] if available.
Strain ID/Accession number	The unique identifier assigned to the specific strain or sequence of the recorded organism, if available.
Database code	The code used by the database the original record was derived from, if available.
Compound name	The chemical name given to the compound in the publication, if available.
SMILES	The Simplified Molecular Input Line Entry System (SMILES) line notation that represents chemical structures using short ASCII strings, if available.
Pathway	Chemical pathway defined by NPClassifier[97] if available.
Superclass	Chemical superclass defined by NPClassifier[97] if available.
Class	Chemical class defined by NPClassifier[97] if available.
Bioactivity	General bioactivity type and detailed bioactivity results including the IC50 or MIC values where possible.
Location	General descriptive location.
Latitude	Latitude in decimal degrees if available.
Longitude	Longitude in decimal degrees if available.
Depth (m)	Depth of sample collection in meters if available.
DOI	The Digital Object Identifier (DOI) of the publication from which the record was derived.
Chemical Novelty	If the compound was deemed chemically novel (N) or not (-) at the time of publication.
Additional information	Any additional information, for example other DOIs that have recorded the compound previously.
Environment	A description of if the sample was collected from the pelagic or benthos, and any details of the habitat or geomorphological feature from which the sample was derived if available.
WoRMS	If the recorded taxonomic classification is (Y) or is not (N) featured in the WoRMS catalogue[98].

Table 2. A summary of the curated interdisciplinary database that describes the abundance, distribution, chemistry, taxonomy, phylogeny, and environment of chemical novelty and bioactivity in the deep sea by 2024.

Data Collection	Count	Description
Total Data Entries	2 909	A database of all deep-sea bioactivity and chemical novelty records (compounds and extracts).
New Compounds	2 197	Recorded as "novel" or "new" at the time of publication (77 % of compounds recorded).
Bioactive Compounds	1 870	Compounds that have shown any form of positive bioactivity result, i.e., a positive half-maximal inhibitory concentration (IC50) or minimum inhibitory concentration (MIC), irrespective of strength (65 % of records).
Bioactive Extracts	30	Compounds are not defined.
**Chemistry**		
Chemical Compounds	2 879	Entries defining chemical compounds, extracts excluded.
Chemical Compound Classes	216	447 compounds could not be defined to a class using the NPClassifier platform[97] and open access literature.
Chemical Compound Super Classes	67	338 compounds could not be defined to a superclass using the N NPClassifier platform[97] and open access literature.
Chemical Compound Structure Pathways	8	Alkaloids (474), Amino acids and peptides (259), Carbohydrates (12), Cyanocobalamin (1), Fatty acids (149), Polyketides (903), Shikimates and phenylpropanoids (131) Terpenoids (734).<br>180 compounds could not be defined to a pathway using the NPClassifier platform[97] and open access literature.
**Bioactivity** *single compounds/extracts may exhibit multiple activities; thus 1900 bioactive compounds/extracts generate 2290 recorded bioactive results.		
Antibacterial Compounds	504	
Antiviral Compounds	98	
Antifungal Compounds	136	
Cytotoxic Compounds	849	
Anti-inflammatory Compounds	205	
Antioxidant Compounds	88	
Enzyme activity	175	
Antiparasitic Compounds	34	
Other Bioactivity	201	
**Taxonomy**		
Kingdoms	5	Archaea, Animalia, Bacteria, Chromista, Fungi. 10 records identified to kingdom only.
Phyla	17	Euryarchaeota, Actinobacteria, Aquificae, Arthropoda, Firmicutes, Proteobacteria, Chordata, Cnidaria, Echinodermata, Mollusca, Nematoda,

		Porifera, Foraminifera, Ochrophyta, Ascomycota, Basidiomycota, Zygomycota. 9 records identified to phyla only.
Classes	34	2 records identified to class only.
Orders	71	1 record identified to order only.
Families	144	14 records identified to family only.
Genera	194	1 162 records identified to genus only (40 %).
Species	224	1 711 records identified to species level (59 %).
**Literature**		
Databases	7	MarinLit[4], CMNPD[83], WoRMS[98], NPClassifier[97], Reaxys[95], Chem Spider[96], Time Tree[99].

Figure 1. Cluster plot of deep-sea organisms recorded as sources of structurally new and/or bioactive compounds, generated using DataWarrior v06.02.01 [ref Sander et al., 2015][108] using a skelspheres descriptor with a 80% similarity index. Links and positioning in show similarity of structural scaffolds and displays associated phyla.

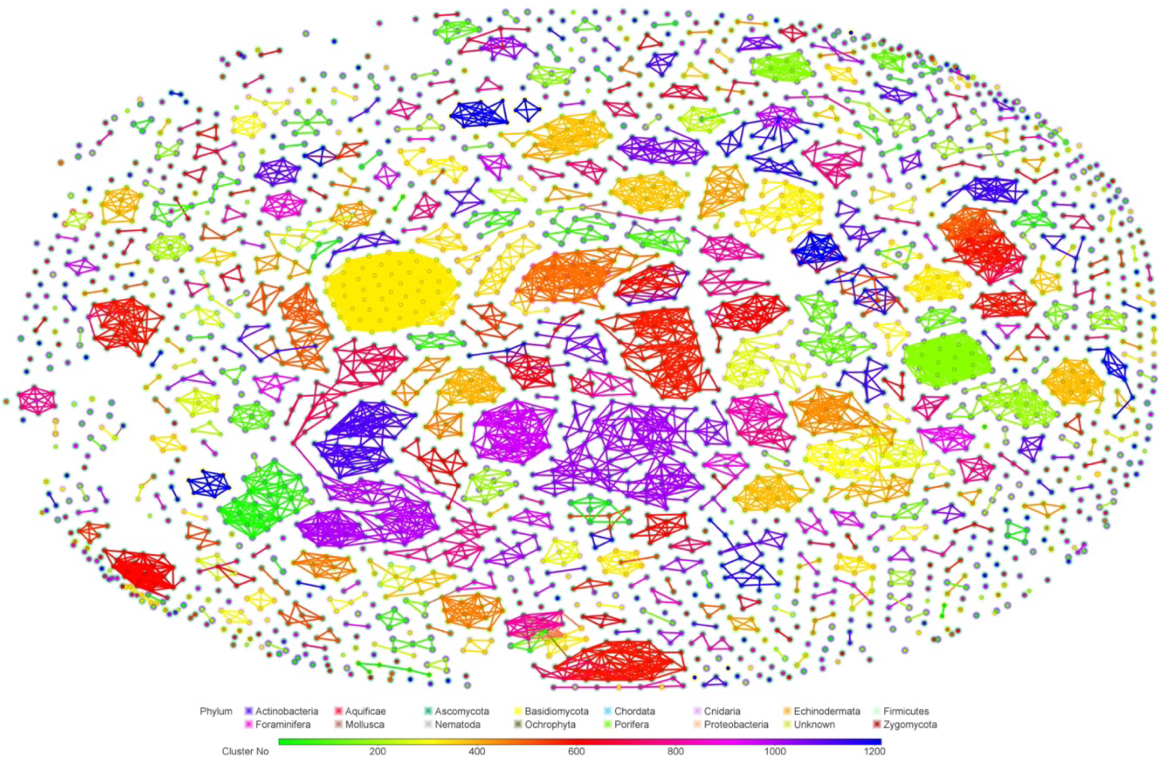

Supplement: Supplementary file 2 — Supplementary Information 2. [file 41598_2026_45398_MOESM2_ESM.pdf]
